# Combined transcranial magnetic stimulation and brief cognitive behavioral therapy for suicide: study protocol for a randomized controlled trial in veterans

**DOI:** 10.1186/s13063-020-04870-6

**Published:** 2020-11-12

**Authors:** Melanie L. Bozzay, Jennifer M. Primack, Hannah R. Swearingen, Jennifer Barredo, Noah S. Philip

**Affiliations:** 1 Department of Psychiatry & Human Behavior, Alpert Medical School of Brown University, Box G-BH, Providence, RI 02912 USA; 2 VA RR&D Center for Neurorestoration and Neurotechnology, Providence VA Medical Center, 830 Chalkstone Boulevard, Providence, RI 02908 USA

**Keywords:** Suicide, Veterans, Transcranial magnetic stimulation, Cognitive behavioral therapy, Neuroimaging

## Abstract

**Background:**

At least 17 veterans die every day from suicide. Although existing treatments such as brief cognitive behavioral therapy (BCBT) have been found to reduce suicide attempts in military personnel, a number of patients go on to attempt suicide after completing therapy. Thus, finding ways to enhance treatment efficacy to reduce suicide is critical. Repetitive transcranial magnetic stimulation (TMS) is a noninvasive technique that can be used to stimulate brain regions that are impaired in suicidal patients, that has been successfully used to augment treatments for psychiatric disorders implicated in suicide. The goal of this study is to test whether augmenting BCBT with TMS in suicidal veterans reduces rates of suicidal ideation, attempts, and other deleterious treatment outcomes.

**Methods:**

One hundred thirty veterans with a suicide plan or suicidal behavior in the prior 2 weeks will be recruited from inpatient and outpatient settings at the Providence VA Medical Center in the USA. Veterans will be randomly assigned to receive 30 daily sessions of active or sham TMS in concert with a 12-week BCBT protocol in a parallel group design. Veterans will complete interviews and questionnaires related to psychiatric symptoms, suicidal ideation and behavior, treatment utilization, and functioning during a baseline assessment prior to treatment, at treatment endpoint, and 6- and 12-month follow-ups. Primary analyses will use mixed effect regressions to examine effects of treatment condition on suicidal behaviors, improvements in psychosocial functioning, and psychiatric hospitalization. Similar models as well as exploratory latent growth curve analyses will examine mediators and moderators of treatment effects.

**Discussion:**

This protocol provides a framework for designing multilayered treatment studies for suicide. When completed, this study will be the first clinical trial evaluating the efficacy of augmenting BCBT for suicide with TMS. The results of this trial will have implications for treatment of suicide ideation and behaviors and implementation of augmented treatment designs. If positive, results from this study can be rapidly implemented across the VA system and will have a direct and meaningful impact on veteran suicide.

**Trial registration:**

This study was registered prior to participant enrollment with ClinicalTrials.gov NCT03952468. Registered on May 16, 2019.

**Trial sponsor contact:**

Robert O’Brien (VA Health Services R&D), robert.obrien7@va.gov

## Background

Suicide is a significant concern among military veterans and has been identified as an important area of emphasis by the US Veteran’s Health System. Approximately 17 veterans die by suicide each day [1]. And, despite advances in the treatment of psychiatric disorders more broadly, suicide rates continue to increase among veterans [1]. Suicide attempt rates are also high and continue to climb among veterans, increasing by 6% from 2005 to 2017 [1]. Efficacious intervention strategies to reduce suicide risk among veterans are thus critically needed, especially during stressful periods (e.g., critical transitions in care, acute crises, etc.) when suicide risk is likely greatest. In this study protocol, we describe novel procedures for a randomized controlled trial (RCT) to augment psychotherapy for suicide with transcranial magnetic stimulation (TMS) among veterans following an acute suicidal crisis.

Despite the public health significance of suicidal behavior, there are few controlled trials to reduce suicidal thoughts and behaviors. Several interventions have been found to reduce suicidal ideation and attempt rates [2–4]. Cognitive behavioral therapy (CBT) has received some of the strongest empirical support for reducing suicide attempts [5, 6]. CBT for suicide is currently the standard of care for suicide treatment in the US Veteran’s Health System. Brief cognitive behavioral therapy (BCBT), a compact version of CBT for suicide, has been found to be efficacious in suicidal and depressed military personnel [5–7]. However, while CBT decreases psychiatric symptoms and suicide, many patients fail to respond to CBT-based therapies and continue to experience high suicide ideation and subsequent reattempts, even in the 1–2 years following treatment when the beneficial effects of treatment should be most apparent (i.e., 14–23% attempting suicide in the active condition) [8, 9]. Thus, finding ways to enhance and boost the efficacy of CBT for suicide is crucial to reduce rates of veteran suicide.

Modulating brain network function is one potential strategy for improving CBT efficacy. Functional brain networks are sets of spatially distributed brain regions that are engaged during similar tasks or conditions [10]. Networks involved in cognitive control enable flexible processing of external and physiological stimuli to suit higher-level goals and adapt to changing contexts [11, 12]. Key BCBT skills, including emotional regulation, are highly dependent on cognitive control. For example, inhibiting reflexive, maladaptive behaviors (e.g., avoidance) in response to physiological distress signals is control-dependent and essential for learning to cope with negative emotions. Notably, cognitive control is often impaired in suicidal patients [13], a factor limiting their ability to engage in psychotherapy.

Our novel approach to augment CBT for suicide is to combine it with neuromodulation of the cognitive control network using repetitive transcranial magnetic stimulation (TMS). TMS is a noninvasive treatment cleared by the US Food and Drug Administration for treatment of pharmacoresistant major depressive disorder, the disorder most prominently implicated in suicide [14]. Growing evidence supports its efficacy in obsessive-compulsive disorder [15], posttraumatic stress disorder [16], and schizophrenia [17]. TMS uses a pulsed magnetic field administered by a coil placed over a targeted brain region to induce neuronal depolarization; repeated administration is hypothesized to induce circuit-level plasticity, impacting function in broader brain networks implicated in psychiatric disorders [18]. Given its minimal side-effects, outpatient administration without anesthesia [19], and the enhanced risk monitoring afforded by daily treatments, TMS may be an ideal adjunctive to CBT for suicide.

The present study design is guided by our overarching hypothesis that adjunctive TMS will increase patients’ ability to manage emotions and respond to stress, thereby enabling patients to more effectively engage in CBT during periods of crisis. Our hypothesis is supported by evidence indicating that brain stimulation can augment related processes such as cognitive control training for depression [20, 21] and fear extinction in posttraumatic stress disorder [22], alongside studies demonstrating the efficacy of TMS to reduce depression symptoms [23], and enhancement of psychotherapy [24]. Additionally, neuroimaging of TMS for depression has found evidence of control and related network modulation [25, 26]. Despite these promising findings, few studies have evaluated whether TMS can reduce suicidal thoughts and behaviors. Our recent review of the literature found that research examining the efficacy of TMS as a treatment for suicide risk is in its infancy [27], with most existing studies limited by their small sample sizes. Notably, at least one study found that active TMS rapidly reduced suicidal thoughts, compared to sham [28], and recent reviews of clinical TMS indicate an empiric reduction in self-reported suicidal ideation [27, 29]. Thus, the extant research highlights the promise of combined cognitive therapy and TMS to reduce suicide, as a novel strategy in suicide intervention research.

Taken together, the fact that suicides have not decreased among veterans despite considerable intervention and prevention efforts indicates that additional strategies to reduce suicide risk are needed. While CBT works for many suicidal patients, a significant portion subsequently attempt suicide (e.g., 14–24%) [6, 30, 31], and thus, finding ways to enhance treatment efficacy and boost the effects of treatment is important. In this protocol, we describe the design of the first RCT to test whether augmenting BCBT for suicide with TMS reduces suicide risk among veterans with a recent suicidal crisis. We have two primary study aims:

### Aim 1: examine whether augmenting BCBT with TMS improves critical suicide outcomes

Our primary hypothesis is that veterans receiving active TMS + BCBT (compared to sham TMS + BCBT) will demonstrate greater reductions in suicidal behaviors. Our secondary hypothesis is that veterans who receive active TMS + BCBT will have reduced suicide attempts and longer time to first attempt after discharge, superior improvements in psychosocial functioning, reduced suicidal ideation severity, and fewer psychiatric hospitalizations/crisis visits during the follow-up period.

### Aim 2: examine treatment moderator and mediator effects

Variables such as diagnosis, comorbidity, and gender will be examined to identify which patients will benefit the most from the addition of active TMS + BCBT versus sham TMS + BCBT. Mediating variables will be examined to determine the psychological mechanisms through which TMS produces its effects on clinical outcomes.

## Methods

### Trial design and study procedure

This is a parallel group, double-blind, two-arm superiority randomized controlled trial conducted in the USA. Participants will be randomly allocated to receive BCBT + real TMS treatment (treatment group) or BCBT + sham TMS (control group). For both groups, approximately 30 sessions of (active or sham) TMS will be administered. TMS sessions will be administered daily on weekdays (approximately 6 weeks in duration). For all participants, TMS sessions will be administered during the 12-week BCBT protocol, in addition to treatment as usual (e.g., pharmacotherapy). Designed as a hybrid of efficacy and implementation research, study outcomes will be measured at baseline, at the treatment endpoint, and at 6-month and 1-year follow-up visits by staff who are blinded to participant condition. Table 1 gives details of assessments and time points. Of note, all items from the WHO Trial Registry Data Set can be found within this protocol.

**Table 1:** Schedule of enrollment, interventions, and assessments

	Baseline	Treatment week												Endpoint	Follow-up	
	Baseline	1	2	3	4	5	6	7	8	9	10	11	12	Endpoint	6 months	1 year
Interventions																
BCBT		X	X	X	X	X	X	X	X	X	X	X	X			
TMS (active or sham)		X	X	X	X	X	X									
Assessments																
Demographics, safety, and treatment																
Demographics	X															
TMS safety form	X															
Treatment History Interview [32]														X	X	X
TMS blinding assessment														X		
Diagnosis and cognitive impairment																
Montreal Cognitive Assessment [33]	X															
Structured clinical interview for DSM-V (Psychiatric and Personality Disorders) [34]	X															
McLean screen for borderline personality disorder [35]	X															
Alcohol use disorders identification test [36]	X													X	X	X
Drug use disorders identification test [37]	X													X	X	X
Suicidal thoughts and behaviors																
Columbia Suicidal Severity Ratings Scale [38]	X													X	X	X
Beck Scale for Suicide Ideation [39]	X													X	X	X
Self-injurious thoughts and behaviors interview [40]	X													X	X	X
Longitudinal interval follow-up evaluation [41]														X	X	X
Beck Hopelessness Scale [42]	X													X	X	X
Comorbid psychiatric symptoms and Functioning																
PTSD Checklist-5 [43]	X	X	X	X	X	X	X							X	X	X
Inventory of depression symptomatology [44]	X	X	X	X	X	X	X							X	X	X
Depression, Anxiety, and Stress Scale	X	X	X	X	X	X	X							X	X	X
Brief symptom inventory [45]	X													X	X	X
World Health Organization Disability Assessment Scale 2.0 [46]	X													X	X	X

*Note. BCBT* brief cognitive behavioral therapy, *DSM-5* Diagnostic Statistical Manual of Mental Disorders-5, *PTSD* post traumatic stress disorder, *TMS* transcranial magnetic stimulation

### Setting

The study will be conducted at the Providence VA Medical Center.

### Ethical approval and trial registration

Ethical approval for the study was obtained from the Providence VA Medical Center Institutional Review Board. This study is registered on the National Controlled Trial registry (NCT03952468) and will be conducted in accordance with the Declaration of Helsinki.

### Participants and recruitment procedures

**Fig. 1:**
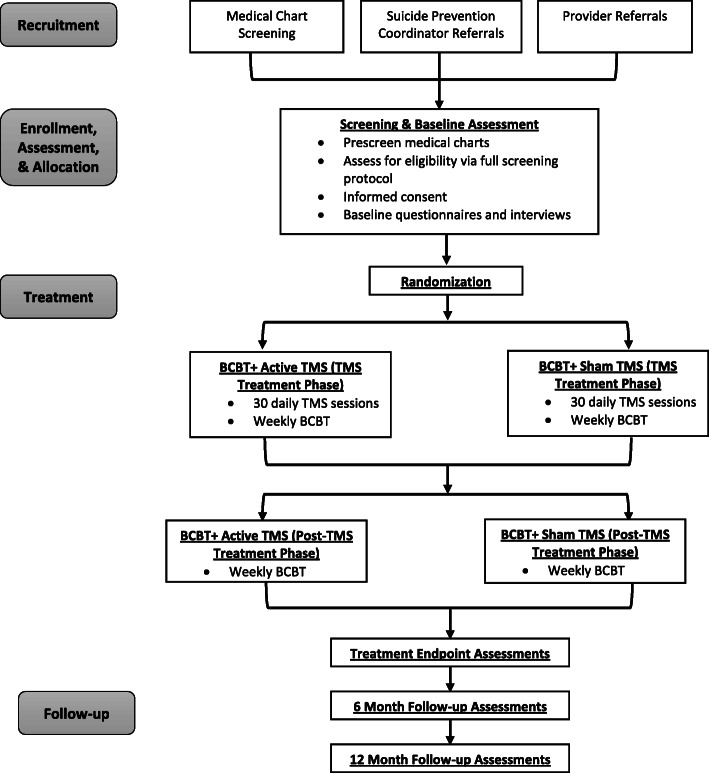
Schematic diagram of the study protocol

Veterans hospitalized for suicide ideation (with a plan to attempt suicide) or suicidal behavior will be recruited from the psychiatric inpatient unit or outpatient clinics at the Providence VA Medical Center. Figure 1 provides a diagram of the participant recruitment. Potential participants will be identified through pre-screening of medical charts of veterans admitted to the inpatient unit, referrals from clinicians (e.g., social workers, attending psychiatrist) on the inpatient unit or outpatient clinics, and referrals from Suicide Prevention Coordinators at the Providence VA. Potentially eligible patients will be approached in-person at the Providence VA and given information about the study by a member of the research staff. Interested patients who endorse suicide ideation or behavior precipitating their inpatient admission, or in the 2 weeks prior to outpatient contact, and who meet TMS safety requirements, will be given the opportunity to provide informed consent. As data from this study may be shared with mechanistic studies drawing from the same participant pool, participants will consent to de-identified data sharing with specific studies as part of their provision of informed consent. All participants will have the right to withdraw their consent for participation at any time. Following consent, participants will complete assessments to further confirm eligibility and establish a baseline level of functioning prior to beginning treatment.

#### Inclusion criteria

Participants will be veterans aged 18–70, any gender, and receiving care at the VA. They must be able to comply with all study-related procedures and visits and be capable of independently reading and understanding study materials and providing informed consent. Participants recruited from the inpatient unit must have been admitted due to suicide ideation with a suicide plan, or a suicide attempt in the prior 2 weeks. Participants recruited from outpatient settings must have attempted suicide or reported a plan to commit suicide in the prior 2 weeks.

#### Exclusion criteria

Participants will be excluded if they meet diagnostic criteria for a primary psychotic (e.g., schizophrenia, schizoaffective disorder) or bipolar I disorder, or past month active moderate-to-severe substance use disorder (with the exception of dependencies on nicotine or caffeine). Medical exclusionary criteria specific to TMS include current significant cognitive impairment (e.g., dementia), current unstable medical condition, prior moderate-to-severe traumatic brain injury, current (or past, if appropriate) significant neurological disorder, or a lifetime history of seizures (except febrile seizures of infancy), central nervous system tumors, stroke, or cerebral aneurysm. Moreover, participants with certain medical conditions that may render TMS administration unsafe, such as having a cardiac pacemaker, or implanted device or metal in the brain, cervical spinal cord, or upper thoracic spinal cord, will be excluded from the study. Female veterans who are pregnant or planning to become pregnant during the study or are of childbearing potential and do not agree to a consistent use of a measure of birth control during the TMS portion of the study will also be excluded from participation.

#### Feasibility of recruitment and retention

This study will build on recruitment and retention strategies used by the investigative team across clinical trials of TMS in veterans [47] and cognitive therapy for severely depressed and suicidal inpatients [48–51]. In our prior TMS research in veterans, more than 90% of participants completed the intensive daily TMS treatment protocol [47]. In our suicide prevention study (Veterans Coping Long Term with Active Suicide Program), we recruited and retained over 100 high-risk veterans throughout the course of treatment, with most returning for multiple follow-up assessments (80% at 3 months). These data support the feasibility and acceptability of our treatment protocol for patients.

#### Randomization

Participants will be randomized to condition via an urn randomization strategy [52, 53]. Urn randomization is a biased coin technique, which randomly assigns veterans of a given subgroup to treatment conditions while systematically biasing randomization to balance treatment conditions on select variables. Here, randomization will be stratified along the following variables related to suicidal behavior or intervention response: admission for suicide attempt vs. suicide ideation, number of previous suicide attempts (none vs. single vs. multiple), and gender (male vs. female). The data analyst for this study will generate a reference document containing the number of participants to be randomized into the treatment conditions in these strata. This reference document will be input to the randomization feature in Red Cap which will ensure that the correct number of participants by strata is randomized per condition.

Participants will be randomized prior to the start of treatment. Each participant will be randomly assigned to one of the treatment groups via the Red Cap computer program by inputting strata features for each patient into Red Cap. Only study team members responsible for ensuring the integrity of the TMS condition (e.g., staff who switch the TMS active and sham coils and do not administer assessments or treatment) will be un-blinded. Each participant’s resulting treatment condition will be recorded in a participant randomization key stored on a secured computer folder accessible only by un-blinded treatment staff.

### Intervention

#### Intervention procedure

Following randomization, participants will complete a 12-week course of BCBT in conjunction with 30 sessions of daily (active or sham) TMS. Prior to each BCBT session, participants will complete the Beck Scale for Suicide Ideation (SSI) [39] to obtain weekly ratings of suicide risk. Symptoms of depression and post-traumatic stress disorder will be measured every 5 sessions of TMS. After completing TMS treatment, participants will finish the remaining BCBT sessions. The intervention will be administered in addition to treatment-as-usual (e.g., pharmacotherapy).

#### BCBT

Participants will receive a standard course of BCBT [7] (12 weekly individual therapy sessions; the first session is 90 min and the remaining eleven sessions are 60 min in duration) provided by a VA mental health counselor. The therapist will have experience and training in cognitive behavioral therapy principles and be trained and supervised weekly by the creator of the BCBT treatment to meet fidelity standards set forth in the BCBT treatment manual. Therapy is divided into three phases: orientation, skill focus, and relapse prevention. Phase one (orientation) includes creating a model of how suicide functions for the patient, developing a safety plan, enhancing treatment motivation, and developing basic emotion regulation skills. Phase two (skills focus) centers on the consolidation of emotional regulation, problem-solving, mindfulness, and cognitive appraisal skills. Phase three (relapse prevention) continues with skills learned in phase two but with a focus on skill generalization and maintenance. Participants will be asked to complete weekly homework tasks to practice skills learned in each therapy session. Therapists will follow the structure and format outlined in the brief cognitive behavioral therapy manual [54] and attend weekly supervision and clinical consultation with one of the therapy developers. Sessions will be audio recorded for subsequent ratings of treatment fidelity. Study therapists will be blind to the treatment condition of each participant.

To limit heterogeneity of therapy administration, participants will be asked to temporarily refrain from other individual psychotherapy, particularly psychotherapy that is CBT-based, while receiving BCBT. However, they will be instructed to continue with all other usual mental health care (i.e., continue prescribed mental health medications and working with their mental health providers).

#### TMS

Participants will receive 6 weeks of daily TMS (i.e., active or sham). Prior to receiving TMS, participants will complete a motor threshold determination to determine the neurostimulation threshold to be used during treatment. The motor threshold is defined as the amount of energy administered via a coil that is required to induce movement in the contralateral hand in at least 50% of stimulations. A separate coil system is used for motor thresholds to reduce accidental unblinding. After the motor threshold is determined, each participant will begin the TMS treatment protocol.

TMS stimulation will use a triple-blind stimulation procedure as implemented in prior randomized controlled trials [47]. Prior to each TMS session, an unblinded study member will assure the setup of either an active or a sham coil according to the participants’ randomization code. Blinded study staff will administer stimulation treatment under the supervision of a blinded attending physician, and participants will not know whether they are receiving active or sham TMS. The sham stimulation coil for this study was selected because it administers sensations to the scalp that are indistinguishable from those experienced during active stimulation (e.g., [50]). To assess the integrity of the blind, participants will be asked which treatment condition they believe they were in at the end of the TMS treatment protocol. TMS treatment (both active and sham) will be delivered using a 70-mm cooled coil via a MagStim Super Rapid 2 + 1 system. Intermittent theta burst TMS will be used for this study; this option was selected because a) it is cleared by the US Food and Drug Administration for pharmacoresistant major depression, and b) each session takes approximately 3 min, thus easing the combination of stimulation and therapy. The (active or sham) coil will be positioned over the left dorsolateral prefrontal cortex using individual scalp landmarks (i.e., Beam/F3 method) [55]. We will use the coil to deliver intermittent theta burst stimulation to the treatment site for 600 pulses at 120% of motor threshold; if participants are unable to tolerate this intensity, we will utilize theta burst delivered at 80% for 1800 pulses (following guidelines from other research in this area [47]).

#### Safety and adverse events

Several procedures are in place to monitor participants’ safety throughout the intervention period. Prior to each TMS session, study staff will verify patients’ medication adherence, assess for any substance use, and query about potential side effects, to ensure ongoing medical safety for TMS. As veterans in our sample are high-risk for suicide, consistent with good clinical practice guidelines in the VA system, participants will be screened for imminent suicide risk at the beginning of each TMS and BCBT session. When clinically indicated, the attending psychiatrist or a licensed therapist will meet individually with the participant to assess risk and identify emergency services if needed. Any adverse events will be reported immediately to the Providence VA IRB and included in the annual IRB report.

#### Discontinuation procedures

Participants will be discontinued from treatment if they choose to withdraw from the study, experience serious symptoms that may be related to the treatment (e.g., seizure, manic episode), and/or cease to be eligible for treatment (e.g., initiate or increase substance use in ways that contraindicate TMS administration). Given the high-risk nature of this patient population, when discontinued from the study, participants will meet with a member of the study staff to discuss alternative treatment options and potential referrals. Research staff will also contact other members of the participant’s treatment team at the VA system to notify them that the participant is no longer receiving care from the study, to facilitate continuity of care. No other information about the participant’s involvement in the study will be disclosed. Unexpected serious adverse events may require unblinding if the information is needed to make informed decisions about the study continuation or patient care. We will report expected and unexpected adverse events in the publication of the results of the trial.

### Measures

Self-report questionnaires and interviews will be administered across the course of the study (at baseline, treatment endpoint, and 6- and 12-month follow-ups) by staff trained and supervised by a licensed clinical psychologist. Assessments will measure key domains of interest (e.g., treatment utilization and history, diagnosis, suicidal thoughts and behaviors, comorbid psychiatric symptoms, and functioning). See Table 1 for a description of the assessment schedule for this protocol.

#### Record review

Study staff will manually extract electronic health record data from participants’ VA medical charts. Data will be extracted to cover the period from baseline through 1 year from the start of the intervention. Study staff will extract information pertaining to health services utilization, medication management and changes, and mental health diagnoses to be used in data analyses. Death by suicide will be captured by review of medical records and death records.

### Study outcomes and mechanisms

#### Primary outcome

The primary outcome (see Table 2) is suicidal events (a composite of all actual, interrupted, and aborted suicide attempts measured by the Columbia Suicide Severity Ratings Scale and Longitudinal Interval Follow-Up Evaluation interviews; CSSRS) at treatment endpoint. Secondarily to this primary outcome, we will examine effects of treatment condition on suicidal ideation severity, number of weeks of active suicidal ideation, suicide attempts, and time to first suicide event after discharge (measured by the Columbia Suicide Severity Ratings Scale and Longitudinal Interval Follow-Up Evaluation interviews; CSSRS). Subsequent time points are considered secondary outcomes.

**Table 2:** Primary and secondary outcomes of the study

Outcome	Domain	Specific measurement	Metric	Method of aggregation	Time point
**Primary**					
Suicidal events	Suicide attempts	CSSRS	Difference in score at a time point	Total score (count)	Endpoint, 6 months, 12 months
Ideation severity	Suicidal ideation	CSSRS	Difference in score at a time point	Total score	Endpoint, 6 months, 12 months
Ideation duration	Suicidal ideation	LIFE	Difference in number of weeks of active suicidal ideation at a time point	Total score (count)	Endpoint, 6 months, 12 months
Suicide attempts	Suicide attempts	CSSRS	Difference in score at a time point	Total score (count)	Endpoint, 6 months, 12 months
Time to first suicide event	Suicide attempts	LIFE	Difference in number of days/weeks from discharge to first event	Total score (count)	Endpoint, 6 months, 12 months
**Secondary**					
Psychosocial function	Function	WHODAS	Total score on scale	Total score	Endpoint, 6 months, 12 months
Number of crisis visits	Crisis visits	THI and record review	Number of visits	Total score (count)	Endpoint, 6 months, 12 months
Number of psychiatric hospitalizations	Crisis visits	THI and record review	Number of hospitalizations	Total score (count)	Endpoint, 6 months, 12 months

#### Secondary outcomes

Besides the primary outcomes, this study will examine multiple secondary outcomes (see Table 2). We will examine improvements in psychosocial functioning (measured by the World Health Organization Disability Assessment Schedule 2.0; WHODAS) and the number of crisis visits and psychiatric hospitalizations (measured by the Treatment History Interview (THI) and record review) measured at treatment endpoint and follow-up assessments.

#### Mediating variables

We will also examine whether improvement in particular processes explains the impacts of treatment conditions on our outcomes of interest. In particular, we will examine changes in suicide risk factors (e.g., hopelessness, global functioning) and associated psychiatric symptoms (e.g., depressive symptoms). These variables will be measured using the self-report questionnaires and interviews collected during the endpoint and follow-up assessments, as noted in Table 1.

### Statistical analysis

#### Data collection

During data collection, several processes will be employed to improve data quality and completeness. Protocols will be developed to describe the procedures that should be completed at each study visit. Research staff will document the completion of each aspect of visit procedures and data collected on case report forms that will be developed by the investigative team. Before the end of each visit, the study staff will review all self-report and interview forms for completeness and ask participants to answer any remaining questions.

#### Data management

To protect participants’ confidentiality, participant data will be labeled using a unique participant identification code that contains no personal identifiers. Paper data will be stored securely in locked file cabinets, and electronic data on VA secure servers accessible only by study staff. Access to participant-identifiable information and data will be limited to researchers included in this study protocol.

#### Data quality

Study staff will employ several strategies to promote data quality, including double data entry, and range checks for data values during study analyses. Interview assessments will be audio-recorded and double-rated for inter-rater reliability purposes and to resolve discrepancies in coding. BCBT sessions will be audio recorded for subsequent ratings of the fidelity of sessions to the BCBT manual. Fidelity ratings will be conducted for 25% of therapy sessions (with sessions randomly selected) by an independent rater based on fidelity checklists provided in the BCBT manual and regularly conducted throughout the course of the clinical trial. Where appropriate, providers not meeting fidelity standards will be supported through further training and additional case supervision time to meet fidelity standards.

#### Data monitoring

A Data Safety Monitoring Board (DSMB) will be responsible for making recommendations to the Principal Investigators regarding changes to the risks and benefits of the study, including recommendations to discontinue new patient enrollments or discontinue the study. The DSMB will be independent from the sponsor and competing interests. The DSMB members will include the study investigators, a suicide prevention expert, a medical expert in TMS, and an expert in statistical analysis. Investigator members will have a reputation for objectivity, absence of conflict of interest (and appearance of the same), and knowledge of clinical study methodology. The DSMB will meet every 6 months to review study progress and any concerns. Participants will be contacted and notified/re-consented in the event that there are important protocol modifications made that impact their participation in the study after being enrolled. All data will be stored for a minimum of 6 years following the end of the trial, in accordance with VA policy.

#### Planned analyses

##### Preliminary analyses

All data analyses will be conducted in the latest version of R Studio. Preliminary analyses will include descriptive statistics to examine the distributional and psychometric properties of the variables (e.g., normality, internal consistency). Variables will be transformed to achieve normality if necessary. We will also examine post-inclusion attrition by comparing study completers to dropouts on sociodemographic variables, baseline characteristics, and length of admission data to determine if they differ systematically. Preliminary analyses may also include analyses of adverse events, progress of recruitment and retention, treatment fidelity across conditions, and quality markers as the study progresses. In keeping with the intention to treat principle, missing data will be handled with multiple imputations with assumptions checked with sensitivity analyses [56–58]. Our main analysis will not adjust for covariates other than the baseline value of the outcome and design factors (balancing factors used in the urn randomization procedure) under the assumption that randomization produced groups balanced on measured and unmeasured confounders.

##### Interim analysis and early stopping of treatment

We will conduct one interim analysis when we have enrolled 32 persons per group (~ 50% enrollment) to assess our primary aim. We will suggest consideration of early stopping to the DSMB if either group shows an effect on the primary outcome using O’Brien-Fleming stopping bounds [59]. The analyst conducting this analysis will be blind to study condition.

##### Effects of treatment (aim 1)

We will conduct several analyses to examine whether augmenting BCBT with TMS improves suicide-related outcomes. The analyst conducting all of these analyses will be blind to treatment condition. To test our *primary hypothesis* (e.g., reduced suicidal behavior), we will conduct an ANCOVA-type mixed effect regression model [60]. In this model, we will regress an outcome composite score of the number of suicidal behaviors (e.g., summation of instances of suicide death, attempt, interrupted or aborted attempts, and suicidal preparatory behavior, consistent with successful approaches to measuring suicidal behavior in other trials [61, 62]) at each time point (treatment endpoint, 6, and 12 months post-baseline) on baseline suicidal behavior, design factors, treatment group, dummy variables for time (baseline as reference), and interactions of treatment group by time. We will use random intercepts and an exchangeable error covariance structure to accommodate non-independence of observations owing to the repeated measures design. The effect of TMS as an adjunctive to BCBT will be tested with the main effect of the treatment group, and the interactions of the treatment group with time will test the maintenance of gains at treatment endpoint, 6, and 12 months. We will also use a Cox proportional hazards model analysis framework to examine these factors in relation to time to first suicide attempt over 12 months.

We will conduct several analyses to test the Aim 1 *secondary hypothesis* (e.g., superior improvements in psychosocial functioning and fewer hospitalizations during follow-up for active versus sham treatment). First, similar to primary hypothesis analyses, we will conduct a repeated measures ANCOVA using a generalized linear mixed effect model, with global functioning (e.g., WHODAS score), and suicide ideation (e.g., C-SSRS score) as outcomes. Second, we will use a negative binomial regression to examine treatment effects on the overall number of crisis visits and psychiatric hospitalizations during each follow-up period.

##### Treatment mediating and moderating effects (aim 2)

To test mediator and moderator effects of treatment, we will conduct exploratory latent growth curve analyses to compare the trajectory of change for primary, secondary, and tertiary outcomes (i.e., linear ascending, descending, or quadratic) between treatment groups. We anticipate including moderator variables in these models (e.g. diagnosis, comorbidity, gender), which may identify patients who will benefit more or less to the addition of active TMS to BCBT. We also will investigate mediating variables to determine the mechanisms through which TMS produces its effects on clinical outcomes. The analytic approach will be similar to that described for the primary and secondary aims, although in addition to the time-point effect indicators, we will examine models with more parsimonious time structure (e.g., linear, piecewise linear, negative exponential) and then attempt to describe factors that predict variations in important components of the implied change trajectory. We will use generalized linear mixed effect models and compare alternative time bases using information criteria before adding covariates, mediators, and moderators. Treatment effect mediation will be evaluated by comparing treatment effects estimated before and after adjusting for the main effect of putative mediators. Moderation effects will be examined as three-way interactions of the putative moderator, treatment assignment, and time.

#### Sample size and power analysis

Approximately 130 veterans will be recruited for this study (65 per treatment group). According to Lehr’s equation [60], this sample size powers this study to detect a medium-sized difference (Cohen’s *d* = 0.5) on our primary outcome (a composite of suicidal events) between treatment groups. This effect size magnitude was selected because it is likely to describe effects of minimal clinical significance or practical importance while being robust to expected attrition (< 10% of attrition among Veterans in our prior intensive TMS studies). This effect size is informed by prior stimulation studies reporting effect sizes of *d* = 0.5 on reductions in depressive symptoms [47]. We note that other studies have found even larger (*d* = 0.9) effect sizes of TMS used with medications [63]. We selected improvement in depressive symptoms as a reference for our power analyses because reductions in depressive symptoms and suicide ideation were highly correlated in our pilot work [27]. We used simulations to estimate the minimum detectible differences on our secondary outcomes (i.e., suicide attempts, suicidal ideation severity, weeks of active suicidal ideation, time to first suicide event). Assuming that 21% of the sample will experience a suicide attempt over 12 months follow-up, if the cumulative risk of suicide attempt is 4.5% over 12 months in the TMS + BCBT group (hazard ratio = 0.19), we will have 81.1% power to detect such an effect using a type-I error rate of 5% in a Cox proportional hazards model framework.

### Dissemination

In the final 6 months of the trial, we will create a task force consisting of the investigators on the team, the local suicide prevention coordinator, and other key stakeholders focused on dissemination of research findings. Study results will be disseminated through conference presentations, publication of results in refereed journals, and the development of a fact sheet describing the preliminary findings of the intervention, to be circulated within the VA system and relevant conferences. Authorship will be granted to members of the study team who contribute substantively to the conceptualization and/or writing of manuscripts. No use of professional writers is planned.

## Discussion

The rate of suicides has not decreased among veterans despite considerable intervention and prevention efforts. As such, there is a critical need for innovative, efficacious strategies to reduce suicide risk, and to understand how treatments reduce suicide risk, to inform subsequent intervention strategies. The RCT described in this protocol represents an important step towards these goals. It will provide critical information regarding whether augmenting BCBT for suicide with TMS reduces suicide risk among a high-risk patient sample of veterans, as well as information about treatment mediators and moderators that can inform future applications of this treatment design.

To our knowledge, this study will be the first clinical trial conducted in the USA evaluating the utility of adding TMS to BCBT to reduce suicide behavior and ideation. If our hypotheses are confirmed, the addition of TMS to BCBT could improve treatment outcomes and decrease suicide ideation and related behaviors. Because TMS reduces symptoms of major depressive disorder [14] and PTSD [16], implementation of this design could potentially alleviate symptoms of comorbid disorders in veterans treated for suicide. Critically, TMS is already available in many Veterans Affairs hospitals across the USA. As such, if positive, results from this study can be rapidly implemented across the VA to have a direct and meaningful impact on Veteran suicide and related symptomatology.

Additional strengths of the design include the presence of an active treatment component in both arms of the study, and implementation of the RCT in a group at highest risk for suicide, military Veterans. This design ensures that all participants in the study receive empirically supported BCBT for suicide. Our study will also provide data about the feasibility of implementing this combined treatment strategy during the high-risk period following an acute suicidal crisis. As such, this study has potential to inform the development of “best practices” for subsequent treatment approaches in this unique, at-risk group.

Several potential challenges of this study design merit consideration. There may be difficulties ensuring adequate recruitment, as the reasons for admission to a psychiatric inpatient unit vary from suicidal crises to dementia. Should we encounter this complication, we will identify veterans in outpatient treatment settings with suicidal crises in the prior 2 weeks. We have developed partnerships with the VA’s Suicide Prevention Coordinators, specialized staff within the VA system who work closely with Veterans at the highest risk of suicide, to facilitate this recruitment approach. In addition, there are potential concerns with attrition due to psychosocial issues (e.g., transportation difficulties) faced by veterans considering suicide. To mitigate these to the extent possible, we will connect Veterans in our study with VA resources (e.g., transportation services) when appropriate. Because substance use is a common comorbidity in this patient population, we utilize broad inclusion criteria related to substances; only those with severe substance use disorders are excluded, with attention to use that prevents safe use of stimulation. Finally, while there are several different TMS approaches, we chose theta burst TMS because it is the fastest and most efficient method available. At nearly 10 times faster than standard TMS (3 min vs. 37.5 min for standard and theta burst TMS, respectively), theta burst provides the opportunity to reach the maximum number of veterans in an eventual clinical roll out.

Taken together, this protocol provides a framework for novel, neuroscience-informed psychosocial treatments. The results of the trial will have implications for the application of neuromodulation to treat suicide risk in conjunction with evidence-based psychotherapy and will provide some of the first information about mediators and moderators of improvement in suicide treatment. As such, these results also have the potential to inform a line of research aimed at optimizing psychosocial treatment for suicide risk and to move us closer to personalized, precision approaches to treatment in this area.

### Trial status

Participant recruitment and data collection for this study began in November 2019. The protocol for this study is IRB-approved and up-to-date (IRB-019-036, Version 1.6, last updated 4/15/2020). Data collection will be completed after all 130 participants have completed the treatment course and follow-up assessments (expected 2022, pending COVID19).

## Data Availability

Not applicable.

## Abbreviations

ANCOVA: Analysis of covariance
BCBT: Brief cognitive behavioral therapy
CBT: Cognitive behavioral therapy
C-SSRS: Columbia Suicide Severity Rating Scale
DSM-5: Diagnostic Statistical Manual of Mental Disorders-5
DSMB: Data Safety Monitoring Board
PTSD: Posttraumatic stress disorder
RCT: Randomized controlled trial
SPIRIT: Standard Protocol Items: Recommendation for Interventional Trials
SSI: Scale for Suicide Ideation
TBS: Theta burst stimulation
THI: Treatment History Interview
TMS: Transcranial magnetic stimulation
VA: Veterans Affairs
WHODAS: World Health Organization Disability Assessment Schedule
